# Utility evaluation of the services provided in international transportation section of East Azerbaijan Province

**Published:** 2019-08

**Authors:** Mohammad Mirzakhani

**Affiliations:** ^*a*^ Department of Development Sociology, Planning and Development Research Institute, University of Tabriz, Tabriz, Iran.

## Abstract

**Background:**

International transportation is one of the main Sections of economy, services of which are used directly and indirectly. Drivers are considered as one of beneficiaries of this section and their comments are of great importance in evaluation of utility of transportation services. Hence, the main research question of the present paper is that how drivers evaluate utility of International Transportation Section services of East Azerbaijan. The aim of this study was being informed of services utility in Jolfa and Norduz border terminals. -Estimation of servicing gap in Jolfa and Norduz border terminals.

**Methods:**

Using survey and questionnaire, 219 local and foreign drivers were interviewed to determine utility of international transportation services of East Azerbaijan. SPSS was employed for data analysis. According to the variables’ measurement level, frequency distribution, diagram, center-oriented statistics and scattering were used in descriptive section and in inferential section correlation coefficient test and averages differences were employed to test the research hypotheses.

**Results:**

Findings of the study indicated that servicing gap of the province border terminals are in road services, transportation companies’ services, border terminals services, medical services, welfare complexes services and police services, respectively. Inferential results indicated that there is a significant correlation between degree of importance and satisfaction from services and from among the above mentioned services, only transportation companies’ services and medical services had significant difference in Jolfa and Norduz border terminals. Other findings, also, indicated that transportation companies’ services, medical services and police services have significant difference in Iran and foreign countries and also border terminals services have significant difference based on transit and non-transit nature of the cargo.

**Conclusions:**

Findings of the study indicated gap in services utility of International Transportation section in East Azerbaijan and hence, the paper’s suggestion is that transportation planners of the province take more attempts in line with improvement of services provided in Jolfa and Norduz border terminals.

**Keywords:**

Services utility, Servicing gap, International transportation, East Azerbaijan Province, Jolfa and Norduz border terminals

